# Rupture of Hepatocellular Carcinoma after Transarterial Chemoembolization followed by Massive Gastric Bleeding

**DOI:** 10.1155/2018/4576276

**Published:** 2018-06-04

**Authors:** Kazuhiro Nishida, Alan Kawarai Lefor, Tomohiro Funabiki

**Affiliations:** ^1^Department of Emergent and Critical Care Medicine, Saiseikai Yokohamashi Tobu Hospital, 3-6-1, Shimosueyoshi, Tsurumi, Yokohama-city, Kanagawa 230-8765, Japan; ^2^Department of Surgery, Jichi Medical University, 3311-1 Yakushiji, Shimotsuke, Tochigi 329-0498, Japan

## Abstract

**Introduction:**

Transarterial chemoembolization (TACE) is the first-line therapy for patient with unresectable hepatocellular carcinoma (HCC). Although TACE is a generally safe procedure, major complications can be occurred. We describe a patient with rupture of HCC after TACE followed by gastric bleeding.

**Case Presentation:**

An 81-year-old man presented with worsening epigastric pain. He had been diagnosed with multiple HCC with nonalcoholic steatohepatitis and underwent TACE 19 days previously. A contrast enhanced computed tomography (CT) scan of the abdomen showed rupture of an HCC. He was treated nonoperatively and discharged on hospital day 18. Five weeks after TACE, he was emergently admitted with massive hematochezia and shock. A contrast enhanced CT scan demonstrated extrinsic gastric compression by an HCC lesion with extravasation of contrast into the stomach. Emergent upper gastrointestinal endoscopy showed a bleeding gastric ulcer with extraluminal compression which was successfully controlled by hypertonic saline-epinephrine injection. Due to tumor progression, he was discharged for palliative care and died six weeks after TACE.

**Conclusion:**

Rupture of HCC is a life-threatening complication after TACE with mortality rates up to 50%. After treatment of a ruptured HCC, extragastric compression and bleeding can occur due to direct compression by a primary lesion or intraperitoneal dissemination.

## 1. Background

Transarterial chemoembolization (TACE) is the first-line therapy for patients with an unresectable hepatocellular carcinoma (HCC) and hepatic metastases. Although TACE is a generally safe, major complications such as tumor rupture, liver abscess, bile leak, hepatic failure, gastrointestinal hemorrhage/ulceration, and pulmonary embolism can occur [1–5]. The major complication rate is reported to be 2.1-2.5% with a mortality rate up to 16.7% [2, 4]. Especially for the patients with an HCC larger than 10 cm, the major complication rate is as high as 4.9% [3]. The incidence of ruptured HCC after TACE is very low, with few published reports. In the 1990s, Liu reported 391 patients who underwent 1443 sessions of TACE, with rupture of an HCC occurring in only six patients (1.5% per patient and 0.4% per procedure) [5]. Two decades later, Tu reported on 1120 patients who underwent 2863 sessions of TACE. Six patients suffered rupture of the HCC (0.5% per patient and 0.2-0.4% per procedure) and two of them died [2]. HCC rupture after TACE is rare but can be a fetal complication.

## 2. Case Presentation

An 81-year-old man presented with abdominal discomfort and distention. His medical history was remarkable for hypertension and type 2 diabetes mellitus. He and his family denied alcohol abuse. Physical examination revealed hepatomegaly without jaundice, ascites, or hepatic encephalopathy. Laboratory data included platelet count 101,000/mm^3^, total bilirubin 0.9 mg/dl, AST 33 IU/L, ALT IU/L, and PT-INR 1.15. The serum AFP and PIVKA-II levels were 1081.0 ng/ml and 43 mAU/ml. Serologic tests for hepatitis B and C virus were negative. The diagnosis of nonalcoholic steatohepatitis with Child-Pugh A liver cirrhosis was made and four HCC lesions were found in segments II, VI, and VIII on the imaging. The largest one was located near the liver capsule in segment II measuring 6.5 cm in diameter. The other three lesions were less than 2 cm with one in segment VI and two in segment VIII. Segmental TACE with epirubicin and iodized oil was performed and he was discharged uneventfully.

**Figure 1:**
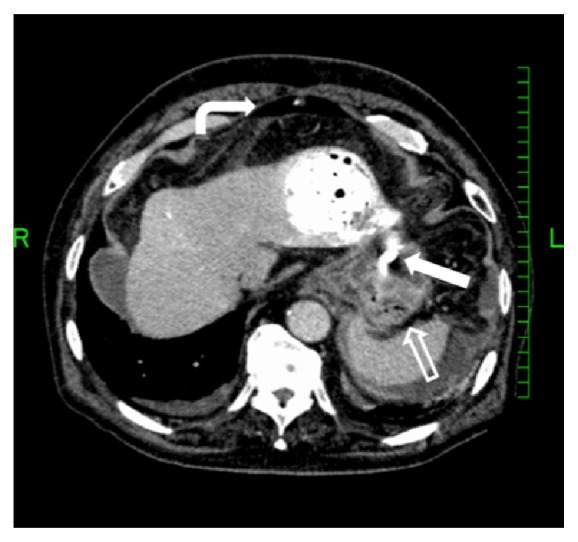
A contrast enhanced computed tomography scan of the abdomen showed iodized oil (arrow) and intraperitoneal free (curved arrow) air with a rupture of the HCC in segment II into the peritoneal cavity adjacent to the gastric wall (open arrow).

He was doing well until he developed abdominal pain 15 days after TACE which was gradually getting worse emergency transport to the hospital. His temperature was 37.7°C, blood pressure 102/41 mmHg, and pulse rate 79/minute. On physical examination, the abdomen was distended and hard to palpation without rebound tenderness. His hemoglobin was 12.6 g/dl. A contrast enhanced computed tomography (CT) scan of the abdomen showed iodized oil and intraperitoneal free air with a rupture of the HCC in segment II into the peritoneal cavity adjacent to the gastric wall (Figure 1). Extravasation of contrast medium was not seen. Emergent upper gastrointestinal endoscopy confirmed no gastric mucosal lesions or a site of perforation. Without evidence of septic shock or hemorrhage, surgical drainage and transcatheter arterial embolization (TAE) are considered less effective. He was treated nonoperatively with piperacillin and tazobactam. Although an abdominal abscess formed, he was discharged on hospital day 18 with continued antimicrobial therapy.

**Figure 2:**
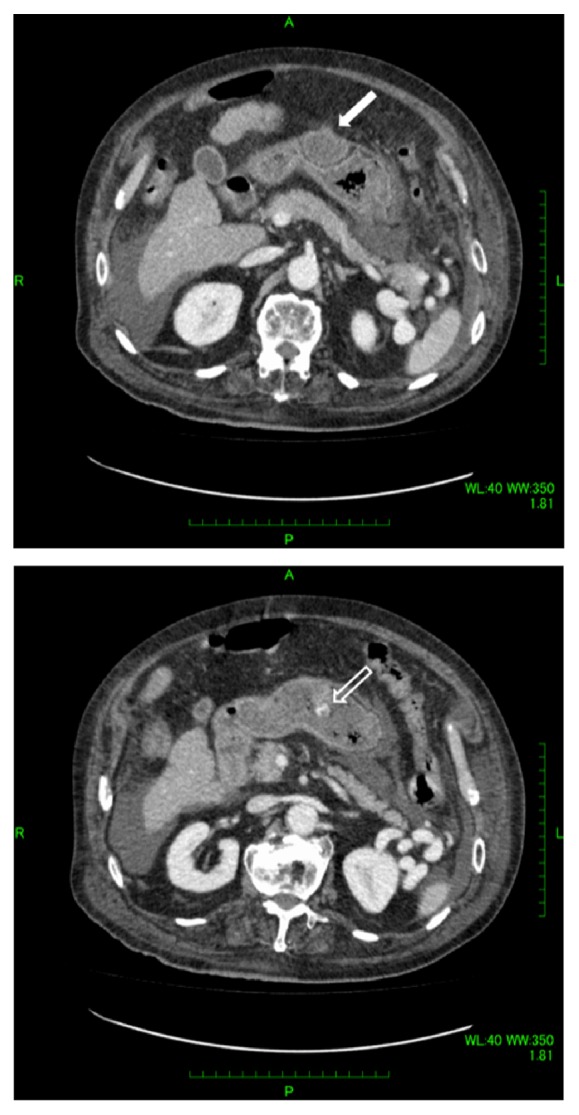
A contrast enhanced computed tomography scan demonstrated gastric extraluminal compression by hepatocellular carcinoma (arrow) and extravasation of contrast medium into the stomach (open arrow).

**Figure 3:**
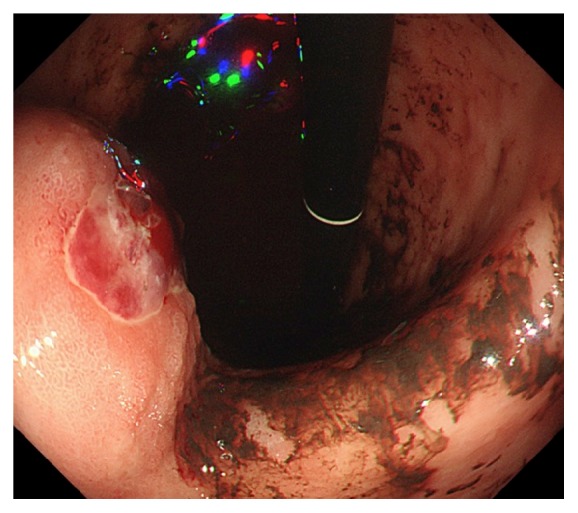
Emergent upper gastrointestinal endoscopy showed a submucosal tumor with central ulceration located on the anterior wall of the gastric body, corresponding to extraluminal compression by the hepatocellular carcinoma.

**Figure 4:**
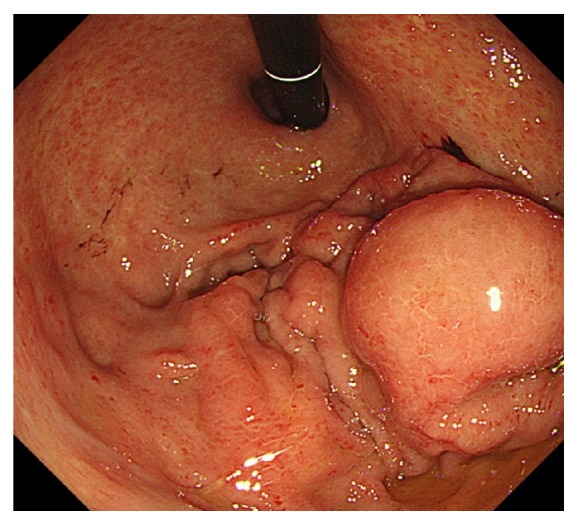
Another submucosal tumor was found in the gastric fundus without ulcer formation.

Five weeks after undergoing TACE, he was readmitted with hematochezia and hemorrhagic shock. The hemoglobin level was dropped to 6.6 g/dl. A contrast enhanced CT scan demonstrated gastric extraluminal compression by an HCC lesion with extravasation of contrast medium into the stomach (Figure 2). Emergent upper gastrointestinal endoscopy showed a submucosal tumor with central ulceration located on the anterior wall of the gastric body, corresponding to extraluminal compression by a HCC (Figure 3). The hemorrhage from the ulcer was successfully controlled by hypertonic saline-epinephrine injection. Another submucosal tumor was found in the gastric fundus without ulcer formation (Figure 4). The patient's condition stabilized and he was discharged for palliative care and died six weeks after undergoing TACE.

## 3. Discussion

The incidence rate of ruptured HCC after TACE is low with few cases reported. An extensive search was conducted (http://www.pubmed.com) for articles related to this topic, using the following search terms: “ruptured hepatic carcinoma” and “TACE” or “transarterial chemoembolization.” A total of 21 previously reported patients were identified and are summarized in Table 1 [2, 6–16].

Fourteen patients were reported from Asia where it is known that hepatitis C virus infection is relatively common. The age ranges from 28 to 90 with a mean of 60 years old. Male gender was 17/20, except for one patient (Number 6 in Table 1) reported as a female by Tu and as a male by Jia [2, 6]. Fourteen of 20 patients had a tumor over 10 cm in diameter. Some case reports suggest that a large tumors increase the risk of rupture [2, 6, 7, 15]. The cause of rupture after TACE is unknown, but fragility of the tumor wall and increased intratumoral pressure are thought to contribute to this complication. Occlusion of the feeding artery leads to necrosis of the tumor which results in wall fragility. Necrosis also leads to increased intratumoral pressure and secondary infection, especially by anaerobic gas forming organisms. In the present patient, the tumor size was 6.5 cm but located near the liver surface. Thin normal liver parenchyma might easily rupture if the tumor wall becomes necrotic with an increase in the intratumoral pressure. An anaerobic infection may have played an important role in the rupture because gas was seen in the tumor and intraperitoneally on the CT obtained at admission in the same location that an abscess later formed.

Treatment for rupture of an HCC after TACE rupture is either nonoperative or exploratory laparotomy. In the last 20 years, TAE has been increasingly used to control bleeding. If there are no signs of peritonitis or active bleeding; nonoperative therapy may be the treatment of choice. If concomitant hemorrhage is evident and hemostasis is necessary, TAE may be better tolerated than laparotomy. The interval between TACE and rupture has been reported 4-6 hours to 5-7 months. A shorter interval seemed to be associated with shorter survival. Since the mortality rate reached 52.4% (11/21) in previously reported patients who suffered HCC rupture, this is a high mortality complication after TACE.

None of the patients with a ruptured HCC had gastric compression or gastrointestinal bleeding except for the present patient. Generally, extragastric compression is rare. Chen et al. performed endoscopic ultrasonography on 55 patients with extragastric compression and found five with malignant etiologies, one of which was HCC [17]. Direct compression by a primary lesion may have resulted in gastric wall extension with erosion which progressed to bleeding.

## 4. Conclusion

HCC rupture is a life-threatening complication after TACE with a mortality rate up to 50%. After treatment of HCC rupture, extragastric compression with subsequent gastric bleeding can occur due to direct compression by a primary lesion or disseminated disease.

## Figures and Tables

**Table 1:** Previously reported cases of HCC rupture after TACE.

No.	Author	Year	Country	Age	Gender	Etiology	Cirrhosis	Tumor size (cm)	Location	Interval	Treatment	Outcome	Cause of death,
1	Tu [2], Jia [6]	2016, 2013	China	45	M	HBV	Present	9	Right	10	Conservative	Died	N.A
2				61	M	N.A.	Absent	13	Right	6	TAE	Alive	
3				53	M	HBV	Present	11	Right+Left	7	Conservative	Died	N.A.
4				57	M	HBV	Absent	14	Right+Left	9	TAE	Alive	
5				64	M	N.A.	Absent	16	Right+Left	17	Conservative	Alive	
6				67	F/M	N.A.	Absent	16	Right+Left	13	Conservative	Alive	
7	Singh Bhinder [7]	2015	USA	67	M	alcohol, HCV	Present	4	VII	1	TAE	Alive	
8	Park [8]	2011	Korea	52	M	HBV, alcohol	Present	12.3	VII	30	Conservative	Died	Hepatic failure, more than 1 year after TACE
9	Bruls [9]	2011	Belgium	78	M	alcohol	Present	7	II, IV	3 weeks	Conservative	Died	Tumor rupture, 2 month after TACE
10	Ritter [10]	2011	Germany	74	M	alcohol	Present	16	III, IVb	14 hours	Conservative	Died	Few hours after tumor rupture
11	Sun [11]	2010	China	28	F	N.A.	N.A.	13	Right	1 month	TAE	Alive	
12				42	F	N.A.	N.A.	11	Right	3	TAE	Alive	
13				83	F	N.A.	N.A.	14	Right	5 month	TAE	Died	Respiratory failure, 1 week after rupture
14				51	M	N.A.	N.A.	7	Right	16 hours	TAE	Alive	
15				47	M	N.A.	N.A.	10	Right	7 months	TAE	Alive	
16	Nawawi [12]	2010	Malaysia	66	M	alcohol	Present	3.5	N.A.	N.A.	N.A.	Died	Tumor rupture, 2 month after TACE
17	Reso [13]	2009	Canada	90	M	N.A.	N.A.	11	Right	4 hours	Conservative	Died	Respiratory failure, 16 days after TACE
18	Reichman [14]	2009	USA	53	M	HBV	Present	6	VII, VIII	6 hours	Laparotomy	Died	Shortly after tumor rupture
19	Battula [15]	2007	UK	61	M	N.A.	Present	11	Right	2	Laparotomy	Died	Tumor rupture, 2 days after TACE
20				69	M	N.A.	Present	13	Right	24	Conservative	Alive	
21	Yeh [16]	2002	Taiwan	45	M	HCV	Present	N.A.	IV-VIII	2 month	Laparotomy	Died	Hepatic failure, one month after surgery

HBV: hepatitis B virus: HCC: hepatocellular carcinoma: HCV: hepatitis C virus: TAE: transarterial embolization: TACE: transarterial chemoembolization: USA: the United States of America: UK: United Kingdom.
